# Antiresorptive Activity of *Bacillus*-Fermented Antler Extracts: Inhibition of Osteoclast Differentiation

**DOI:** 10.1155/2013/748687

**Published:** 2013-02-10

**Authors:** Sik-Won Choi, Seong-Hee Moon, Hye Jeong Yang, Dae Young Kwon, Young-Jin Son, Ri Yu, Young Su Kim, So I. Kim, Eun Jeong Chae, Sang-Joon Park, Seong Hwan Kim

**Affiliations:** ^1^Laboratory of Translational Therapeutics, Pharmacology Research Center, Bio-Organic Science Division, Korea Research Institute of Chemical Technology, P.O. Box 107, Yuseong-gu, Daejeon 305-600, Republic of Korea; ^2^Food Functional Research Division, Korean Food Research Institute, Sungnam 463-746, Republic of Korea; ^3^Department of Pharmacy, Sunchon National University, Suncheon 540-742, Republic of Korea; ^4^Department of Histology, College of Veterinary Medicine, Kyungpook National University, Sankyuk-dong, Buk-gu, Daegu 702-701, Republic of Korea; ^5^Korean Medicine Biofermentation Co. Ltd., Seoul 137-894, Republic of Korea

## Abstract

Antlers have been traditionally used for thousands of years as a natural product with medicinal and pharmaceutical properties. In developing healthy foods, *Bacillus*-mediated fermentation is widely used to enhance the biological activity of nutrients in foods. Recently, fermentation was shown to enhance the osteogenic activity of antlers. This study aimed to elucidate the antiresorptive activity of *Bacillus*-fermented antler and its mode of action. We found that *Bacillus*-fermented antler extract strongly inhibited osteoclast differentiation by downregulating the expression and activity of nuclear factor of activated T cells, cytoplasmic 1 (NFATc1). This extract also inhibited the activation of phospholipase C**γ**2 (PLC**γ**2), a signaling molecule that could regulate NFATc1 transcriptional activity. This suggested that *Bacillus*-fermented antler extract could inhibit PLC**γ**2-NFATc1 signaling required for bone resorption and cell fusion. Consequently, *Bacillus*-fermented antler extract might benefit osteoclast-related disorders, including osteoporosis; furthermore, it may improve gastrointestinal activity.

## 1. Introduction

Bone homeostasis is maintained by a tight balance between osteoclast-mediated bone resorption (or destruction) and osteoblast-mediated bone formation. An imbalance induced by the overactivation of osteoclast differentiation (or osteoclastogenesis) can lead to a variety of bone metabolic diseases, including osteoporosis. Therefore, a promising strategy for treating patients with osteoporosis is to inhibit osteoclast differentiation.

For thousands of years, antlers have been traditionally used in Asian countries, due to their many medicinal and pharmaceutical properties. The combination of beneficial factors such as several amino acids, sulfated-glycosaminoglycan, sialic acid, gangliosides, neutral lipids including cholesterol ester, phospholipids including phosphatidylcholine, lecithin, and polyamines in antler extracts has been suggested to exhibit the several salutary activities in humans. Apparently, the antler is dried and is used powdered or in tea form for a wide variety of health remedy and health maintenance purposes in Asian countries such as China and Korea. Additionally, antler extracts are sold in Asian countries as the type of functional food.

The beneficial role of antler extracts in the prevention and/or treatment of osteoporosis has not been studied well, but several studies have reported their beneficial action in bone metabolism and in the experimental condition of osteoporosis; antler extracts have exhibited antiosteoporotic activity by increasing the proliferation of osteoblasts and the expression of bone matrix protein [1]. Chondroitin sulfate isolated from deer antler tips has been shown to upregulate the gene expression of bone-specific proteins in a human osteoblastic cell line [2]. Furthermore, anti-resorptive activities of antler extracts, antler blood, collagen, and glue were also reported in several studies [3–5]. These results indicated the dual antiosteoporotic activity of antler to stimulate bone formation and inhibit bone resorption.

Moreover, fermentation caused an enhancement of antler osteogenic activity [1]. Among bacteria used for fermentation, *Bacillus* strains are wildly used to develop healthy foods, because they enhance the biological activity of nutrients in foods. The representative *Bacillus*-fermented healthy foods are Korean food “Cheonggukjang” and Japanese food “Natto.” Indeed, *Bacillus subtilis* has been sold commercially around the world as a nutritional supplement in healthy foods [6]. Fermented antler exhibited bone anabolic activity, but it has not been elucidated whether antler fermented with *Bacillus subtilis* has antiresorptive activity. Therefore, this study aimed to evaluate the anti-resorptive activity of antler fermented with *Bacillus subtilis* and its mode of action in osteoclast differentiation.

## 2. Materials and Methods

### 2.1. Antlers

Antlers (*Cervus canadensis E*.) and fermented antlers were provided by Korean Medicine Biofermentation Co. Ltd. (Seoul, Korea). Briefly, antlers were purchased from the DaeSeong Elk Farm (Kangwon, Korea). Samples comprised a combination of three sections (40% top; 30% middle; 30% base). The samples were homogenized and stored at −20°C.

### 2.2. Bacteria and Culture Conditions

*Bacillus subtilis* strain K-11 was isolated from soybeans by Professor Dong-Hyun Kim (College of Pharmacy, Kyung Hee University, Seoul, Korea). Bacteria were cultured in Tryptic Soy Agar or Broth (Difco, KS). Strains were inoculated in Tryptic Soy Broth and grown at 37°C for 1 day.

### 2.3. Preparation of Antlers and Fermented Antler Extracts

The nonfermented antler extract was prepared by soaking antlers in distilled water (1.5 kg/30 L), followed by autoclaving at 121°C for 20 min, refluxing at 100°C for 3 h, filtering twice with nonwoven fabric, and lyophilizing at −55°C for 4 days. Fermented antler extract was prepared by inoculating the antler sample with 1% (v/v) *Bacillus subtilis* strain K-11 after the antler sample had been soaked and autoclaved in distilled water (800 g/30 L). The mixture was incubated in a fermenter (Seobong Biogen, Kangwon, Korea) at 37°C for 5 days. Then, the fermented antler extract was filtered twice with non-woven fabric and lyophilized at  −55°C for 4 days.

### 2.4. Osteoclast Differentiation

Bone marrow cells were obtained from 5- to 8-week-old male ICR mice by flushing femurs and tibias in *α*-MEM supplemented with antibiotics (100 units/mL penicillin, 100 *μ*g/mL streptomycin; Hyclone, UT). Bone marrow cells were cultured for 1 day in 10-cm culture dishes with *α*-MEM that contained 10% fetal bovine serum (FBS; Gibco, Paisley, UK), antibiotics, and macrophage colony stimulating factor (M-CSF; 10 ng/mL; Peprotech, NJ). Nonadherent bone marrow cells were plated on 9-cm petri dishes and cultured for 3 days in the presence of M-CSF (30 ng/mL). After nonadherent cells were washed out, adherent cells comprised bone marrow-derived macrophages (BMMs). BMMs were induced to differentiate into osteoclasts by culturing (1 × 10^4^ cells/well in a 96-well plate or 3 × 10^5^ cells/well in a 6-well plate) for 4 days in the presence of M-CSF (30 ng/mL) and the receptor activator of nuclear factor kappa-B ligand (RANKL; 5 ng/mL; R&D Systems, MN).

### 2.5. TRAP Staining and Activity Assay

Mature osteoclasts were visualized by staining tartrate-resistant acid phosphatase (TRAP), a biomarker of osteoclast differentiation. Briefly, cells were fixed with 10% formalin for 10 min and 0.1% Triton X-100 for 10 min, and then stained by using the Leukocyte Acid Phosphatase Kit 387-A kit (Sigma, MO). Images were captured under a microscope equipped with a DP Controller (Olympus Optical, Tokyo, Japan). The number of TRAP-positive osteoclasts was counted on the whole area in a well under a microscope. For measuring TRAP activity, cells were fixed with 10% formalin for 10 min and 0.1% Triton X-100 for 10 min. Then, we added 100 *μ*L of citrate buffer (100 mM, pH 5) that contained 50 mM sodium tartrate and 3 mM *p*-nitrophenylphosphate (Sigma) to the fixed cells. After incubation for 1 h, the enzyme reaction mixtures were transferred into new plates containing an equal volume of 0.1 N NaOH. Absorbance was measured at 405 nm with a Wallac EnVision HTS microplate reader (Perkin Elmer, MA). The experiment was performed in triplicate.

### 2.6. Cell Viability Assay

BMMs were suspended in *α*-MEM with 10% FBS and plated in a 96-well plate at a density 1 × 10^4^ cells/well. BMM cells were treated with the fermented antler extract in the presence of M-CSF (30 ng/mL) and incubated for 1 or 3 days. Cell viability was then measured with the Cell Counting Kit-8 (Dojindo Moleculer Technologies, MD) according to the manufacturer's protocol. Measured absorbance was converted to cell number with a standard curve.

### 2.7. Real-Time PCR

Primers were chosen with an online primer 3 design program [7]. The primer sets used in this study are shown in Table 1. Total RNA was isolated with TRIzol reagent (Invitrogen, NY) according to the manufacturer's protocol. First-strand cDNA was synthesized with the Omniscript RT kit (Qiagen, CA) and 1 *μ*g of total RNA, 1 *μ*M of oligo-dT_18_ primer, 10 units of the RNase inhibitor, RNasin (Promega, WI), according to the manufacturer's protocol. Then, quantitative PCR was performed with the Stratagene Mx3000P real-time PCR system and Brilliant SYBR Green Master Mix (Stratagene, CA). The first-strand cDNA was diluted 1 : 10 and 10 pmol of primers were added into each reaction, according to the manufacturer's protocol. The thermocycling protocol consisted of 3 segments. The first segment comprised incubation at 95°C for 10 min to activate the polymerase; the second segment comprised 40 cycles of 94°C for 40 s (denaturation), 53°C for 40 s (annealing), and 72°C for 1 min (extension); the third segment was an incubation at 95°C for 1 min, 55°C for 30 s, and 95°C for 30 s to generate PCR product temperature-dissociation curves (also called “melting curves”). All reactions were run in triplicate, and the data were analyzed with the 2^−ΔΔC_T_^method [8]. Glyceraldehyde-3-phosphate dehydrogenase (GAPDH) was used as an internal standard. The statistical significance was determined by the Student's *t*-test, with GAPDH-normalized 2^−ΔΔC_T_^ values. 

### 2.8. Western Blot Analysis

Briefly, cells were homogenized and centrifuged at 10,000 ×g for 15min. The supernatant was collected to isolate cytoplasmic proteins. Denatured proteins were separated on the SDS-PAGE gels and transferred onto PVDF membranes (Millipore, CA). The membrane was then washed with TBST (10 mM Tris-HCl pH 7.5, 150 mM NaCl, and 0.1% Tween 20) and incubated in the blocking solution, TBST with 5% skim milk. The membrane was probed with the indicated primary antibody, washed three times for 30 min, incubated with secondary antibody (Santa Cruz Biotechnology) conjugated to horseradish peroxidase for 2 h, and washed three times for 30 min. The membranes were developed with SuperSignal West Femto Maximum Sensitivity Substrate (Pierce) and signals were detected in the LAS-3000 luminescent image analyzer (Fuji Photo Film Co., Ltd., Japan). Antibodies against NFATc1 and Actin were purchased from Santa Cruz biotechnology (CA). Antibodies against (p)-ERK, (p)-JNK, (p)-p38, (p)-Plc*γ*2, ERK, JNK, p38, and Plc*γ*2 were obtained from Cell Signaling Technology. All antibodies were diluted 1 : 1000 in TBST with 1% BSA.

### 2.9. Luciferase Activity Assay

Human embryonic kidney 293T cells were plated in a 24-well plate in triplicate. Cells were then transfected with the following reporter plasmids: (1) the plasmid (100 ng/well) with the DNA-binding sequence for the nuclear factor of activated T-cells, cytoplasmic 1 (NFAT) fused to the firefly-luciferase sequence, (2) the plasmid (100 ng/well) with the receptor activator of nuclear factor *κ*B (RANK) sequence, and (3) the plasmid (20 ng/well) with the renilla luciferase sequence in the pGL4 vector. After 6 hrs, the transfected cells were co-treated with RANKL (50 ng/mL) and the fermented antler extract. After 48 h, the transfected cells were lysed with lysis buffer (Promega, Madison, WI, USA), and luciferase activity was measured with a dual-luciferase assay system (Promega). Luciferase activity was normalized to renilla luciferase activity for each sample.

### 2.10. Retrovirus Preparation and Infection

Retrovirus packaging was described previously [9]. In brief, to isolate the retroviral particles, we transiently transfected Plat-E cells (platinum-E retrovirus packaging cell line, Ecotropic, Cell Biolabs, Inc.) with pMX-IRES-GFP, a retrovirus vector with green fluorescence protein (GFP), and the pMX vector with a constitutively active (CA) NFATc1 gene. At 48 h after transfection with Lipofectamine 2000 (Invitrogen, NY), the viral supernatants were collected from the culture media according to the manufacturer's protocol. Next, BMMs were incubated with the viral supernatants in the presence of polybrene (10 *μ*g/mL) for 8 h. The infection efficiency was determined by GFP expression, which was always greater than 80%. After infection, BMMs were induced to differentiate in the presence of M-CSF (30 ng/mL) and RANKL (5 ng/mL) for 4 days.

### 2.11. Statistical Analysis

Each experiment was performed in triplicate and repeated three to five times to confirm the reproducibility of data, and results from one among repeated experiments were analyzed and shown in the figures. Statistical differences were analyzed with the Student's *t-*test and all quantitative values were presented as mean ± SD. A value of *P* < 0.05 was considered significant.

## 3. Results

### 3.1. Fermented Antler Extract Inhibited RANKL-Induced Osteoclast Differentiation

**Figure 1:**
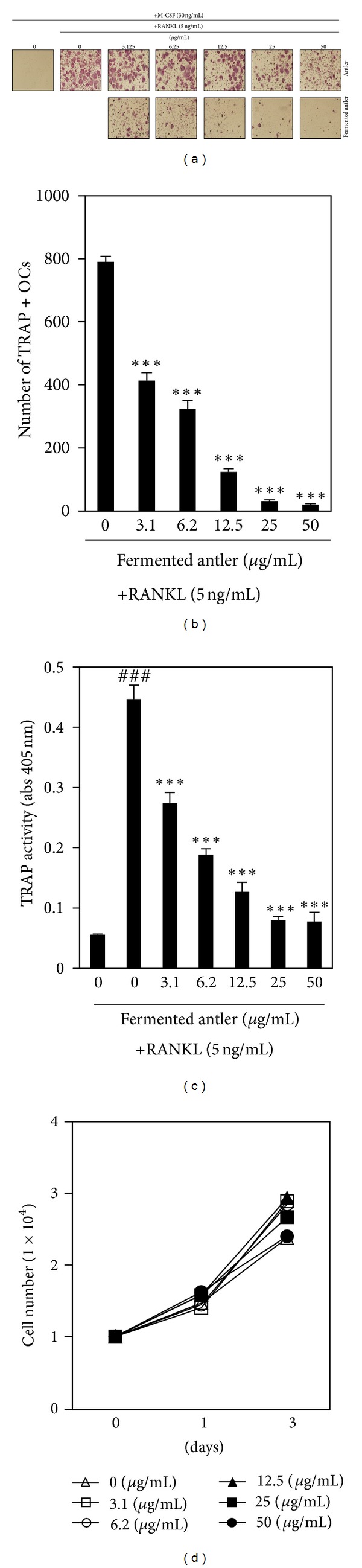
Fermented antler extract inhibits RANKL-induced osteoclast differentiation. (a) BMM cells were cultured for 4 days in the presence of RANKL (5 ng/mL) and M-CSF (30 ng/mL) with antler or fermented antler extract. Osteoclasts were visualized by TRAP staining. (b) The number of TRAP-positive osteoclasts (TRAP+OCs) was counted. ***P < 0.001 (versus “the control”). (c) TRAP activity was measured. ^###^ *P* < 0.001 (versus “the negative control”); ***P < 0.001 (versus “the group treated with RANKL alone”). (d) The effect of fermented antler extract on the viability of BMMs was evaluated by CCK-8 assay. Each experiment was performed in triplicate. Statistical differences were analyzed with the Student's *t-*test and all quantitative values were presented as mean ± SD.

Osteoclast differentiation was evaluated with the RANKL-induced osteoclast differentiation model of primary mouse BMMs. The effect of fermented antler extract was compared to the effect of non-fermented antler extract. We found that, during BMM differentiation into osteoclasts, both antler and fermented antler extracts dose-dependently inhibited the formation of TRAP-positive multinucleated osteoclasts (Figure 1(a)). The inhibitory effect of fermented antler extract on osteoclast differentiation was confirmed by counting the number of TRAP-positive osteoclasts and by evaluating TRAP activity (Figures 1(b) and 1(c)); in a dose-dependent manner, the fermented antler extract significantly inhibited the RANKL-induced formation of TRAP-positive osteoclasts and activation of TRAP. To ascertain that the inhibitory effect of fermented antler extract on RANKL-induced osteoclast differentiation was not due to cytotoxicity *per se*, its effect on cell viability was evaluated. At the concentrations used in this study, the fermented antler extract did not show any cytotoxicity in BMMs (Figure 1(d)).

### 3.2. Fermented Antler Extract Inhibited RANKL-Induced NFATc1 Expression and Its Transcriptional Activity

**Figure 2:**
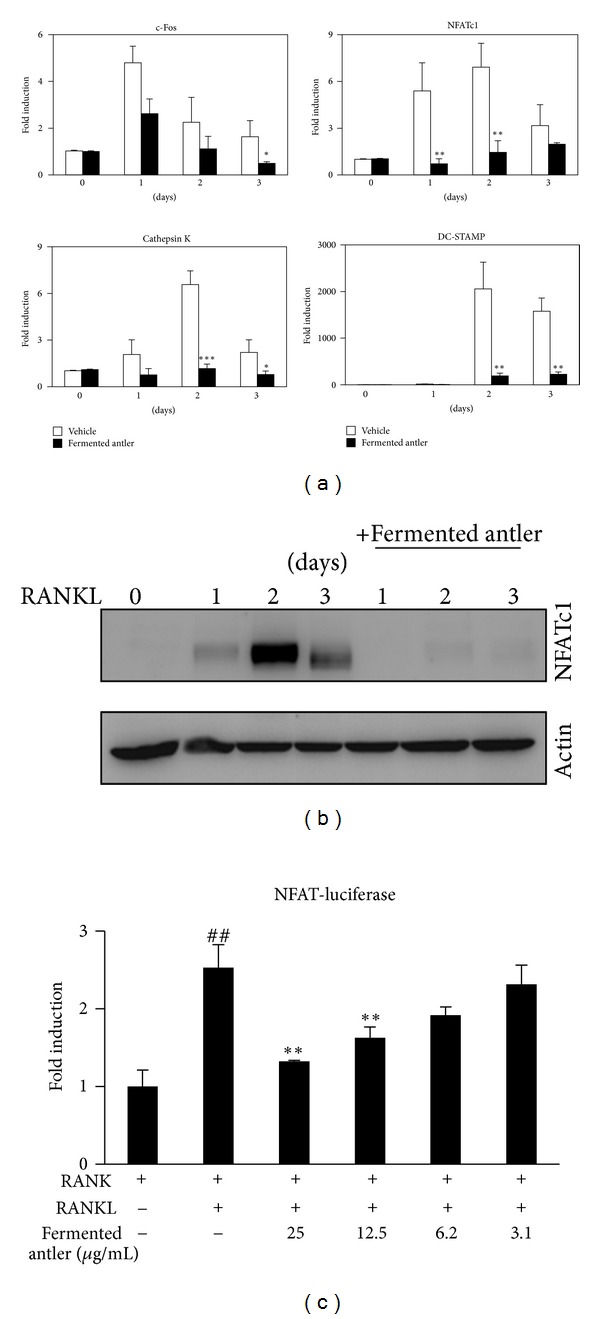
Fermented antler extract suppresses RANKL-induced NFATc1. (a) After pretreated with vehicle or fermented antler extract (25 *μ*g/mL) for 1 hr, BMMs were treated with RANKL (5 ng/mL) for the indicated day, and then the mRNA expression levels were analyzed by the real-time PCR. *P < 0.05; **P < 0.01; ***P < 0.001 (versus “the vehicle control”). (b) The effect of fermented antler extract on the protein expression of NFATc1 was evaluated by the Western blot analysis. Actin was used as the internal control. (c) The effect of fermented antler extract on the transcriptional activity of NFATc1 was evaluated by luciferase activity assay. Activity was expressed as fold induction compared with the activity of NFAT luciferase only. pGL4 renilla luciferase activity was used to normalize the transfection efficiency and luciferase activity. ^##^ *P* < 0.01 (versus “the RANK”); **P < 0.01 (versus “the RANK plus RANKL”). Each experiment was performed in triplicate. Statistical differences were analyzed with the Student's *t*-test and all quantitative values were presented as mean ± SD.

The inhibitory effect of fermented antler extract on osteoclast differentiation was further explored by evaluating the expression of transcription factors. As shown in Figure 2(a), the mRNA expression levels of both c-Fos and NFATc1 were strongly induced by RANKL, but the induction of NFATc1 was significantly inhibited by the fermented antler extract in the early stage of osteoclast differentiation. The expression levels of cathepsin K and DC-STAMP were also elevated with RANKL, but these inductions were significantly inhibited by the fermented antler extract. Furthermore, the RANKL-induced mRNA expression of TRAP was also significantly inhibited by the fermented antler extract (data not shown). 

Western blot analysis revealed that the RANKL-mediated induction of NFATc1 protein was completely inhibited by the fermented antler extract (Figure 2(b)). The NFATc1-luciferase reporter activity assay showed that the RANKL-mediated activation of NFATc1 was dose-dependently inhibited by the fermented antler extract (Figure 2(c)). These results suggested that NFATc1 could be involved in the anti-resorptive activity of fermented antler extract.

### 3.3. Fermented Antler Extract Inhibited NFATc1-Induced Osteoclast Differentiation

**Figure 3:**
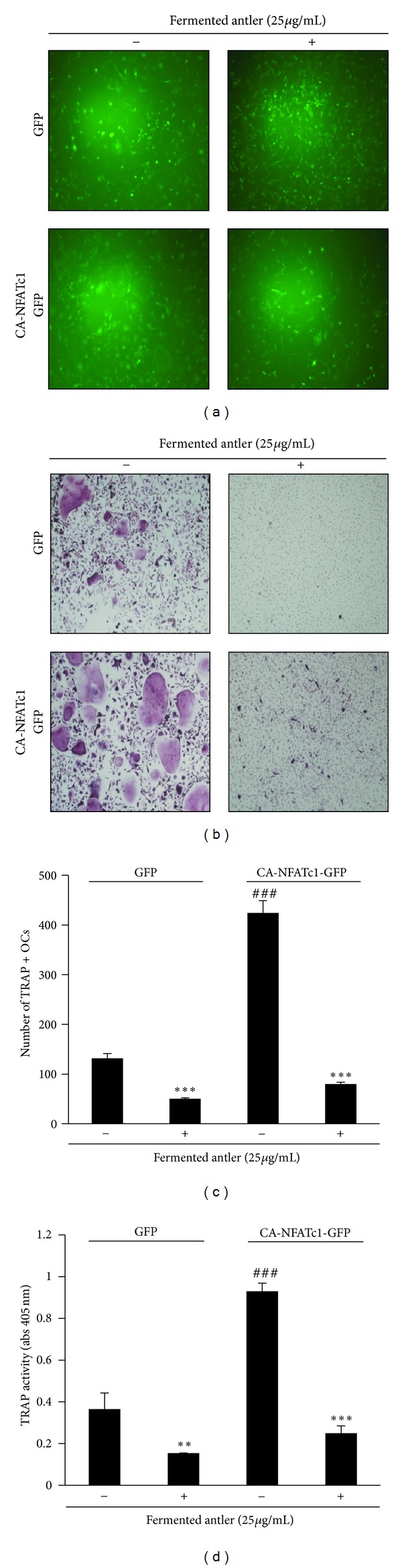
Fermented antler extract suppresses NFATc1-induced osteoclast differentiation. (a) BMMs were infected with pMX-IRES-GFP (GFP) or pMX-IRES-CA-NFATc1-GFP (CA-NFATc1-GFP) for 8 hrs with polybrene (10 *μ*g/mL). The infected BMMs were cultured with M-CSF (30 ng/mL) and RANKL (5 ng) for 4 days in the presence or absence of fermented antler extract (25 *μ*g/mL). After 4 days, cells were fixed, and the GFP expression was visualized under a fluorescence microscope. (b) BMMs were infected with GFP or CA-NFATc1-GFP and then cultured as described in (a). After 4-day culture, mature TRAP-positive osteoclasts were visualized by TRAP staining. (c) TRAP-positive cells (TRAP+OCs) were counted as osteoclasts. ^###^ *P* < 0.001 (versus “the GFP control”); **P < 0.01; ***P < 0.001 (versus “the fermented antler-nontreated group”). (d) TRAP activity was measured at 405 nm. ^  ###^ *P* < 0.001 (versus “the GFP control”); **P < 0.01; ***P < 0.001 (versus “the fermented antler-nontreated group”). Each experiment was performed in triplicate. Statistical differences were analyzed with the Student's *t-*test and all quantitative values were presented as mean ± SD.

We hypothesized that the anti-resorptive activity of fermented antler extract might result from its potential to completely inhibit the expression of NFATc1. We tested this hypothesis by overexpressing NFATc1 in mouse BMMs with a retroviral vector. The infection rates of the retroviral GFP control and the retroviral constitutively active (CA) NFATc1-GFP were similar in either BMMs treated or untreated with the fermented antler extract (Figure 3(a)). TRAP-positive multinucleated osteoclasts were significantly more abundant in BMMs overexpressing NFATc1 than in BMMs treated with the GFP control. However, in the presence of fermented antler extract, the over-expression of NFATc1 could not induce BMM differentiation into osteoclasts (Figure 3(b)). This strong inhibitory activity of fermented antler extract on the NFATc1-mediated formation of TRAP-positive osteoclasts was confirmed by counting the number of osteoclasts (Figure 3(c)) and measuring the activity of TRAP (Figure 3(d)).

### 3.4. Fermented Antler Extract Inhibited RANKL-Induced PLC*γ*2 Activation

**Figure 4:**
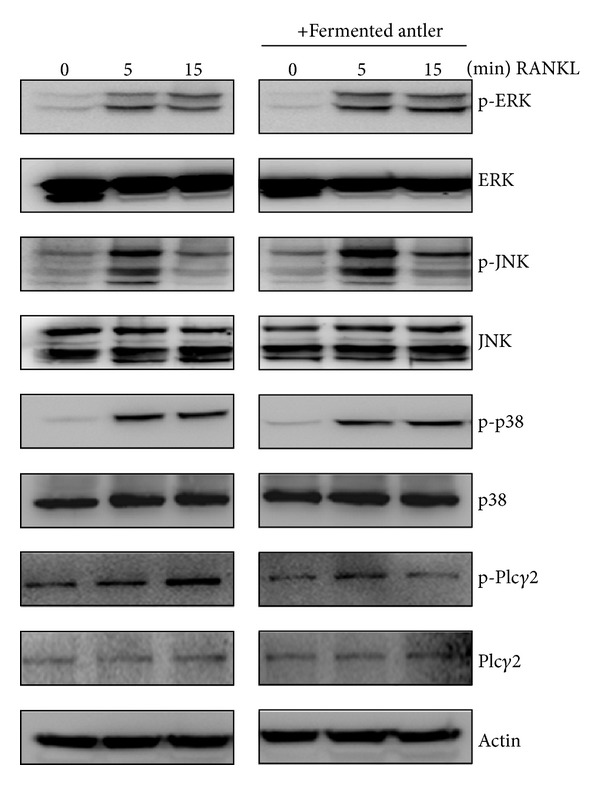
Fermented antler extract inhibits RANKL-induced phosphorylation of PLC*γ*2. BMMs were pretreated with or without fermented antler extract (25 *μ*g/mL) for 1 hr prior to RANKL stimulation (5 ng/mL) at indicated time periods. Then, the protein expression levels were evaluated by Western blot analysis.

To gain insight into the mechanism by which the fermented antler extract might block the NFATc1-related process of osteoclast differentiation, we investigated the effect of fermented antler extract on the activation of signaling molecules involved in osteoclast differentiation. First, we considered MAP kinases, because they are well known to be involved in osteoclast differentiation [10]. As shown in Figure 4, RANKL strongly induced the activation of ERK, JNK, and p38, but those inductions were not inhibited by the fermented antler extract. Next, we focused on the involvement of PLC*γ*2 activation in the anti-resorptive activity of fermented antler extract because PLC*γ*2 activation can evoke a Ca^2+^ signal required for the activation and induction of NFATc1 in osteoclast differentiation [11]. Interestingly, the fermented antler extract inhibited the RANKL-induced phosphorylation of PLC*γ*2. These results suggested that the PLC*γ*2-NFAT signaling axis could be involved in the anti-resorptive activity of fermented antler extract.

## 4. Discussion

There are two main treatment strategies for reducing the incidence of osteoporotic fracture and the reduction of bone resorption with anti-resorptive agents, like bisphosphonates, and the induction of bone formation with anabolic agents, like parathyroid hormone (PTH). PTH is a currently available for stimulating bone formation, but its use is limited by cost and concerns regarding its long-term safety. Thus, anti-resorptive agents have become the therapeutic mainstay for treating osteoporosis. However, the most common anti-resorptive agent like bisphosphonates also carries the risk of side effects such as bisphosphonate-related osteonecrosis of the jaws [12] and atypical femoral fractures [13]. Therefore, there is a strong need for new anti-resorptive agents.

Natural products have historically yielded a variety of therapeutic agents. Generally, healthy nutrients or foods with medicinal properties are both effective and safe for the long-term management of disorders. Recent studies have aimed to identify natural products or healthy foods that can prevent and/or treat osteoporosis, with minimal adverse effects [14, 15]. 

Due to many medicinal and pharmaceutical properties, antlers have been recognized in traditional medicines. Recently, several studies have shown that antler extract and its components exhibited anti-osteoporotic activity. Moreover, fermentation of antlers enhanced their osteogenic activity [1]. However, the anti-resorptive activity of antlers fermented with *Bacillus subtilis* and the mode of action have not been elucidated.

In this study, we found that the antler extract fermented with *Bacillus subtilis* strain K-11 significantly inhibited the RANKL-induced differentiation of BMMs into osteoclasts in a dose-dependent manner. In a previous study, a chloroform extract of deer antler inhibited osteoclast differentiation via suppressing the RANKL-induced activation of ERK [16], but in this study, the fermented antler extract inhibited the RANKL-induced phosphorylation of PLC*γ*2, not those of MAP kinases. 

Transcription factors, c-Fos and NFATc1, were known to play a critical role in the regulation of genes required for osteoclast differentiation. The c-Fos transcription factor, an AP-1 family member, was suggested to be essential for osteoclast differentiation [17], and NFATc1 was shown to rescue osteoclastogenesis in cells that lacked c-Fos [18–20]. Furthermore, the coordinating activation between AP-1 and NFATc1 may play an important role in the regulation of osteoclast-specific genes, like TRAP and cathepsin K [21]. Additionally, the NFATc1-induced dendrite cell-specific transmembrane protein (DC-STAMP) was reported to be essential for osteoclast fusion [22–24]. In this study, fermented antler extract strongly inhibited RANKL-induced NFATc1 expression and its transcriptional activity and furthermore, it also inhibited NFATc1-induced osteoclast differentiation.

It is known that the activation of NFATc1 requires assembly of the RANKL-RANK-MAP kinases and *PLC*⁡*γ*-Ca^2+^ signaling. Previous studies showed that, in osteoclast precursors, RANKL triggered the activations of MAP kinases and PLC*γ*, and those activations consequently induced the activation of transcription factors [10]. In the present study, we suggested that the fermented antler extract could inhibit the signaling axis of PLC*γ*2-NFATc1 during osteoclast differentiation. This suggestion was consistent with findings that the PLC*γ*2-NFATc1 signaling axis could positively regulate RANKL-induced osteoclast differentiation in mice; in that study, PLC*γ*2-deficient mice had an osteopetrotic phenotype and exhibited reduced NFATc1 expression [25]. Furthermore, in this study, the fermented antler extract strongly inhibited the NFATc1-mediated formation of TRAP-positive multinucleated osteoclasts even in BMMs overexpressing a constitutively active NFATc1. These results suggested the possible involvement of PLC*γ*2-NFAT signaling axis in the anti-resorptive activity of fermented antler extract. 

Moreover, the inhibition of NFATc1 expression was expected to reduce NFATc1-regulated gene expression. Previous studies showed that proximal NFAT binding sites played a significant role in the NFATc1-induced gene expression of cathepsin K in response to RANKL [26] and that NFATc1 induced osteoclast fusion by upregulating DC-STAMP [24]. In this study, we found that the fermented antler extract inhibited the RANKL-induced gene expression of cathepsin K and DC-STAMP; these effects were most likely due to the inhibition of NFATc1 expression. 

This study showed that the fermented antler extract strongly inhibited osteoclast differentiation by down-regulating NFATc1 expression and activity. Our results suggested that the mechanism of this inhibition involved the inactivation of PLC*γ*2. The consequence of down-regulating NFATc1 expression was a decrease in the transcription of cathepsin K and DC-STAMP, essential factors for bone resorption and cell fusion, respectively. Further study will be required to identify the components in the fermented antler extract that confer anti-resorptive activity and the detailed *in vivo* experiment should be carried out before its application to humans. Our results finally suggested that *Bacillus*-fermented antler extract might be developed to functional foods or pharmacological agents for preventing and treating osteoclast-related disorders as well as improving gastrointestinal activity [27].

## Figures and Tables

**Table 1:** Primer sequences used in this study.

Target gene	Forward (5′–3′)	Reverse (5′–3′)
c-Fos	CCAGTCAAGAGCATCAGCAA	AAGTAGTGCAGCCCGGAGTA
NFATc1	GGGTCAGTGTGACCGAAGAT	GGAAGTCAGAAGTGGGTGGA
Cathepsin K	GGCCAACTCAAGAAGAAAAC	GTGCTTGCTTCCCTTCTGG
DC-STAMP	CCAAGGAGTCGTCCATGATT	GGCTGCTTTGATCGTTTCTC
GAPDH	AACTTTGGCATTGTGGAAGG	ACACATTGGGGGTAGGAACA
